# The association of *FKBP5* gene methylation, adolescents’ sex, and depressive symptoms among Chinese adolescents: a nested case-control study

**DOI:** 10.1186/s12888-022-04392-2

**Published:** 2022-11-30

**Authors:** Wenyan Li, Wanxin Wang, Wenjian Lai, Xiuwen Li, Liwan Zhu, Jingman Shi, Kayla M. Teopiz, Roger S. McIntyre, Lan Guo, Ciyong Lu

**Affiliations:** 1 Department of Medical Statistics and Epidemiology, School of Public Health, Sun Yat-Sen University, 74 Zhongshan Rd 2, 510080 Guangzhou, China; 2 Mood Disorders Psychopharmacology Unit, University Health Network, Toronto, ON Canada; 3 Department of Pharmacology, University of Toronto, Toronto, ON Canada; 4 Department of Psychiatry, University of Toronto, Toronto, ON Canada; 5 Institute of Medical Science, University of Toronto, Toronto, ON Canada

**Keywords:** Depressive symptoms, *FKBP5* DNA methylation, Sex differences, Social-environmental factors, Nested case-control study

## Abstract

**Background:**

Depressive symptoms among adolescents are a serious health concern around the world. Altered DNA methylation in the FK506 binding protein 5 (*FKBP5*) gene has been reported to regulate stress response, which has been reported to be closely associated with depressive symptoms. However, most of the contributing studies have been conducted among adults and relatively few studies have considered the effect of disparate social influences and sex differences on the DNA methylation of *FKBP5* in persons with depressive symptoms. The present study aimed to test the associations of *FKBP5* DNA methylation and depressive symptoms among adolescents and explore possible sex differences in the foregoing associations.

**Methods:**

This study was conducted using a nested case-control design within a longitudinal cohort study from January 2019 to December 2019. Adolescents aged 12 to 17 years from 69 classes in 10 public high schools located in Guangdong province of China participated in this research. Students with persistent depressive symptoms that reported having depressive symptoms at both baseline and follow-up were treated as the case group, and those without depressive symptoms were randomly selected as the control group. Our study finally included 87 cases and 151 controls. Quantitative methylation analyses of the selected gene were carried out by MassARRAY platform System.

**Results:**

The overall DNA methylation trend of *FKBP5* CpG sites in the case group was lower in comparison to the control group. Compared to healthy controls, lower methylation percentage of *FKBP5*-12 CpG 1 was observed in adolescents with persistent depressive symptoms after adjusting for covariates (case: 0.94 ± 2.00, control: 0.47 ± 0.92; *F* = 5.41, *P* = 0.021), although the statistical significance of the difference was lost after false discovery rate correction (*q* > 0.05). In addition, the hypomethylation of *FKBP5*-12 CpG 1 was approaching significance after adjustment for social-environmental factors (a*OR* = 0.77; *P* = 0.055), which indicated that no independent association was detected between hypomethylation of *FKBP5* CpG sites and persistent depressive symptoms. Furthermore, in the present study, we were unable to identify sex differences in the association of *FKBP5* gene methylation with depressive symptoms.

**Conclusion:**

The decreased methylation level of *FKBP5* was observed in adolescents with persistent depressive symptoms, albeit non-significant after correction for multiple testing. Our results presented here are preliminary and underscore the complex gene-environment interactions relevant to the risk for depressive symptoms.

**Supplementary Information:**

The online version contains supplementary material available at 10.1186/s12888-022-04392-2.

## Background

Depression is a leading cause of disability worldwide, with more than 280 million people affected [1]. It has been reported that adolescence is a very vulnerable period for developing mental health problems [2], and depressive symptoms (DS) show peak incidence in adolescence [3]. In addition, compared to boys, girls are two times more likely to experience DS [4]. According to the Global Burden of Disease study, DS is one of the leading causes of years lived with disability in adolescents[5]. Depressive symptoms in adolescents are considered as a predictive marker for later development of the major depressive disorder (MDD) in adulthood and many other adverse psychosocial outcomes [6], such as suicide attempts [7], anxiety disorders [6], and risky behaviors [8]. However, the underlying molecular mechanisms of adolescent depression and sex differences in the prevalence rate appear complex and remain unclear.

Evidence shows that the dysregulation of the hypothalamic-pituitary-adrenal (HPA) axis is a mechanism responsible for DS as the HPA axis is the major neuroendocrine axis responding to stress via a feedback loop [9]. FK506 binding protein 5 (*FKBP5*) gene is an important negative-feedback regulator of the glucocorticoid receptor (GR) complex [10], which plays a critical role in HPA axis regulation [11, 12]. Emerging evidence suggests that depression involves epigenetic mechanisms [13, 14]. As one of the most common epigenetic modifications, DNA methylation reflects an integrated view of both environmental stress and genetic contribution to depression [15, 16]. Research on methylation of HPA-axis genes has suggested that altered methylation levels of the *FKBP5* gene may have potential as a biomarker of depression [17, 18], however, results concerning methylation levels of the *FKBP5* gene in depressive symptoms are inconsistent. Reduced methylation levels of the *FKBP5* gene have been demonstrated to be associated with depression in some but not all studies [17–19].

Studies of *FKBP5* show support for a significant association between reduced DNA methylation and increased risk of psychiatric disorders among adults, such as post-traumatic stress disorder and depression [17, 18]. However, due to the cross-sectionally designed, causal inferences in such studies are limited. Conversely, some studies reported the opposite relationship [20–22]. For example, Roberts et al. [22] reported that a decrease in DNA methylation at the promoter region of *FKBP5* was associated with a greater treatment response in adults with psychiatric disorders. Moreover, there were also studies that found no association between the degree of DNA methylation in *FKBP5* and depressive symptoms [23–26]. Taken together, the role of *FKBP5* gene expression as causally related to depression is yet to be elucidated. Furthermore, most of these studies have focused on adults with MDD, and whether the *FKBP5* methylation is altered in adolescents with DS remains unknown.

To date, relatively few studies have explored possible sex differences in *FKBP5* methylation among adolescents with depressive symptoms. A better understanding of such differences may provide important insights into mechanisms relevant to the association between *FKBP5* methylation and depressive symptoms, as well as potentially inform prevention and treatment for persons at increased risk. The newest research revealed that DNA methylation is a promising biomarker that may help explain the underlying mechanism for observed sex differences in persons experiencing mental disorders [27].

Sex-specific DNA methylation has previously been studied in different human tissues, such as blood and the brain [27, 28]. A study analyzing sex-differentially methylated positions and regions in human postmortem brain samples reported higher methylation in males than in females at more than half autosomes [27]. However, there is a dearth of studies reporting on sexual differences in *FKBP5* DNA methylation levels in individuals with DS, particularly in adolescence, which has been reported as the typical period of onset for DS.

Therefore, we conducted a nested case-control study among Chinese adolescents which aimed to investigate whether there is a significant association between *FKBP5* DNA methylation and DS after adjusting for multiple social-environmental risk factors. The foregoing analysis also considered social context and environment, which can also predict DS. Furthermore, even less is known about sex differences in the association between DNA methylation level and DS, therefore, our second aim was to test the effect of sex differences in the foregoing association.

## Methods

### Study design and participants

The Longitudinal Study of Adolescents’ Mental and Behavioral well-being Research (LSAMBR) [29, 30] is a school-based cohort study conducted in Guangzhou in south China (Registration No. ChiCTR1900022032). The LSAMBR was designed to observe mental and behavioral health development in students studying in first year of seven junior schools and three senior high schools.

A nested case-control study design was used in the study herein based on the LSAMBR. Data from baseline and one-year follow-up research were collected in January 2019 and December 2019, respectively. A total of 1,976 participants were recruited, with 1,956 participants completing a standardized self-reported questionnaire at baseline assessment (response rate: 98.99%) and 1,836 participants completing a one-year follow-up assessment (retention rate: 93.87%). The inclusion criteria of the LSAMBR were as follows: (a) all students in grades 7 and 10 studying in selected classes; (b) parent and participating student provided written informed consent; exclusion criteria included the following: (a) history of neurological disorders, such as brain injury, encephalitis, and epilepsy; (b) evidence of mental retardation; (c) unable to participate in the study due to physical disease; (d) history of severe psychiatric disorder. In the current nested case-control study, students with persistent DS were treated as the case group if they were less than 18 years old, had blood samples collected at baseline, and were considered as having DS both at baseline and follow-up visit, while those without DS both at baseline and follow-up were randomly selected as the control group. Finally, 87 cases and 151 controls were included for analysis.

The LSAMBR was approved by the Sun Yat-sen University, School of Public Health Institutional Review Board (Ethics Number: L2017060). The objectives and methodology of this study were explained to students and parents/guardians and written informed consent was obtained from them. Participation in this research was voluntary. LSAMBR guidelines and regulations were rigorously followed throughout the investigation.

### Measures

#### Depressive symptoms

Depressive symptoms were measured using the Center for Epidemiologic Studies Depression Scale (CES-D) at baseline and 1-year follow-up. The CES-D is a 20-item self-reported measure of depressive symptoms with good internal consistency and construct validity [31]. Items have a 4-point Likert scale from 0 (“rarely or none of the time”) to 3 (“most or all of the time”). The total score ranges from 0 to 60, with higher scores indicating greater levels of DS. The Chinese version of the CES-D shows satisfactory reliability and validity among Chinese adolescents [32, 33]. In this study, Cronbach’s alpha of this scale was 0.85, exhibiting high internal reliability. We adopted the extensively utilized cut-off point of 28 to identify students with DS [34].

#### Blood collection and DNA preparation

Fasting EDTA blood (5 ml) was collected in the early morning (8:00 to 10:00 am) at the baseline assessment visit, and stored at -80℃ prior to analysis. DNA was isolated from peripheral blood using a DNA extraction kit (BioTeke Corporation, Beijing, China). All the operations were carried out strictly in accordance with the instructions of the kit. The quality and quantity of the extracted DNA samples were examined with a NanoDrop 2000 C spectrophotometer (Thermo Scientific, Waltham, MA, USA). DNA samples meeting the following criteria were considered qualified: the total amount should be > 1 µg; samples had no degradation or DNA contamination; samples had a 260/280 ratio between 1.5 and 2.0.

#### Assessment of DNA Methylation

We used the CpG Island Online Prediction website (http://www.ebi.ac.uk/Tools/seqstats/emboss_cpgplot/) to predict CpG islands in the *FKBP5* promoter region (chr6: 35,728,998–35,729,370 and chr6: 35,729,453–35,729,707) based on the CpG island determination criteria (%GC > 50, length > 200 bp, Obs/Exp CpG > 0.6). PCR primers were designed by using Epidesigner (Agena Bioscience, Inc., San Diego, CA, America). After a comparison of several primer design options, two primers with the best coverage were chosen (#12 and #47, details in Supplementary Material).

Briefly, the detection steps of methylation were as follows. The target gene regions were modified by sodium bisulfate, which converted any non-methylated cytosine residues into uracil, while methylated cytosine residues were unaffected, and then it was amplified by PCR with primers specific for methylated versus unmethylated DNA. The resultant PCR product was inactivated by dephosphorylation using shrimp alkaline phosphatase (SAP). Subsequently in vitro transcription was performed by tagging the reverse PCR primer with the T7 RNA polymerase and the resulting RNA transcripts were specifically cleaved at uracil residues. The resulting cleavage products differed in signal patterns for methylated and non-methylated DNA templates, depending on the sequence changes generated through initial bisulfite conversion, and then were analyzed using MALDI-TOF mass spectrometry. Cleavage product signals with a mass shift of 16 Da (or a multiple thereof) represented methylated DNA fragments, and signal intensity had a relation with the methylation degree, which allowed for proper quantification of DNA methylation. Finally, the methylation of 42 CpG units, encompassing 71 CpG sites, was quantified using the MassARRAY platform (Agena Bioscience, Inc.) [35].

The methylation data were preprocessed and quality controlled, where CpG units with < 80% of available methylation were excluded to ensure that spurious data were not analyzed [36]. In addition, significantly deviating data points (> 3 times the interquartile range (IQR)) were also excluded [37]. A total of 29 CpG units encompassing 47 CpG sites were ultimately qualified for analysis (Table S1).

#### Other information

Demographic variables included sex (1 = boy, 2 = girl), age, body mass index (BMI), family structure, family relationship, household socioeconomic status (HSS), academic performance, smoking (1 = yes, 2 = no), and alcohol consumption (1 = yes, 2 = no) were also collected, which had been reported to be associated with DS in previous studies [4, 38, 39]. BMI was calculated as weight (kg)/ (height (m))². Family structure was divided into three categories, living with “both parents”, “single parent”, and “other relatives”. Family relationship was measured by asking participants’ perceptions of their family relationship (1 = good, 2 = average, 3 = poor). HSS was assessed by participants’ subjective perceptions of their familial financial status (1 = good, 2 = average, 3 = poor). Academic performance was assessed by asking adolescents’ class rank at school (1 = good, 2 = average, 3 = poor).

Morning serum cortisol level was also measured. A separate venous blood sample was collected from participants at 8–10 am to obtain serum, which was collected at the same time points at the baseline visit as the blood samples for methylation analysis. The serum total cortisol level was assayed with the competitive chemiluminescent microparticle immunoassay using the Abbott Architect i2000SR system (Abbott Laboratories, Abbott Park, IL). The assay coefficient of variation (CV) was 4.0–6.2% at low levels and 3.3–4.3% at high levels.

### Data analysis

All statistical tests were conducted using SPSS 25.0 software package (IBM, Armonk, New York, NY, USA) and R Language (V4.0.3). First of all, descriptive analysis with *t*-test and Chi-square test was performed to describe baseline characteristics. Second, for each methylation site, a significant *t*-test was followed up by the univariate analyses of variance using family structure, family relationship, household socioeconomic status, academic performance, and alcohol consumption, as covariates. Third, hierarchical clustering was generated according to the different healthy statuses (persistent DS cases or healthy controls) and sexes (boys and girls). The intensity of the methylation signal was color-coded using the R package “pheatmap” (https://CRAN.R-project.org/package=pheatmap), then the results were visualized as a heatmap. Finally, the univariate and multivariate logistic regression were used to determine the risk factors of DS. Odds ratios (ORs) and 95% confidence intervals (CIs) of OR were calculated. Socio-demographic variables with a *P*-value < 0.05 in the univariate logistic regression analysis were considered for inclusion in the multivariable logistic regression analysis to determine whether the differentially methylated sites were independently associated with persistent DS. Moreover, all logistic regression analyses were stratified by sex. Statistical differences were considered to be significant if the *P* < 0.05. To address the multiple comparisons problem and potential Type I errors, the false discovery rate (*FDR*) [40] was calculated for multiple testing correction. The term of *FDR*-adjusted *P* was indicated by “*q*” and the results were considered as significant when *q* < 0.05.

## Results

### Demographic characteristics stratified by depressive symptoms and sexes

The demographic characteristics of participants were detailed in Table 1. The average participant age was 13.6 ± 1.4 years, ranging from 12 to 17. There were significant differences between the case and control groups in terms of sex, family structure, family relationship, household socioeconomic status, academic performance, and drinking. Adolescents with persistent DS were predominantly girls (64.4%, *P* = 0.013). Adolescents living in a single-parent family (20.7%) or living with others (11.5%), with poor family relationships (12.6%) and economic conditions (8.1%) were more general in the case group compared to controls (*P* < 0.05). In addition, cases had poor academic performance in school (34.5%, *P* < 0.001) and more alcohol consumption (51.8%, *P* < 0.001). Moreover, the total mean serum cortisol level was 230.3 ± 104.0 µg/mL and the mean BMI values were 19.6 ± 3.5 kg/m^2^, which was in the normal range and not statistically different between the persistent DS group and control group.

**Table 1:** Sample characteristics by depressive symptoms and sexes (*N* = 238)

Variables	Total	Depressive symptoms^#^	Controls	$\frac{x^2}{t^\psi}$	*P* ^*^	Boys	Girls	$\psi$	*P* ^*^
	*n* (%)	*n* (%)	*n* (%)			*n* (%)	*n* (%)		
Total	238 (100.0)	87 (36.6)	151 (63.4)			110 (46.2)	128 (53.8)		
Group								6.182	**0.013**
Depressive symptoms^#^	87 (36.6)					31 (28.2)	56 (43.8)		
Controls	151 (63.4)					79 (71.8)	72 (56.2)		
Sex				6.182	**0.013**				
Boys	110 (46.2)	31 (35.6)	79 (52.3)						
Girls	128 (53.8)	56 (64.4)	72 (47.7)						
Age (year, mean (SD))	13.6 (1.4)	13.6 (1.4)	13.6 (1.5)	-0.178	0.859	13.8 (1.5)	13.5 (1.4)	1.589	0.114
BMI (kg/m^2^, mean (SD))	19.6 (3.5)	20.0 (3.7)	19.4 (3.4)	-1.411	0.160	20.1 (4.2)	19.2 (2.8)	1.746	0.083
Cortisol concentration (µg/mL, mean (SD))	230.3 (104.0)	234.4 (111.1)	227.9 (100.0)	-0.465	0.643	231.8 (97.8)	229.0 (109.5)	0.210	0.834
Family structure				12.117	**0.002**			2.716	0.257
Living in a two-parent family	189 (79.7)	59 (67.8)	130 (86.7)			91 (83.5)	98 (76.6)		
Living in a single-parent family	31(13.1)	18 (20.7)	13 (8.7)			10 (9.2)	21 (16.4)		
Living with others	17 (7.2)	10 (11.5)	7 (4.6)			8 (7.3)	9 (7.0)		
Missing data	1	NA	NA			NA	NA		
Family relationship				24.160	**< 0.001**			3.410	0.182
Good	188 (79.0)	54 (62.1)	134 (88.7)			92 (83.6)	96 (75.0)		
Average	35 (14.7)	22 (25.3)	13 (8.6)			14 (12.7)	21 (16.4)		
Poor	15 (6.3)	11 (12.6)	4 (2.7)			4 (3.7)	11 (8.6)		
HSS				9.552	**0.008**			1.694	0.429
Above average	123 (51.7)	37 (42.5)	86 (57.0)			52 (47.3)	71 (55.5)		
Average	106 (44.5)	43 (49.4)	63 (41.7)			53 (48.2)	53 (41.4)		
Below average	9 (3.8)	7 (8.1)	2 (1.3)			5 (4.5)	4 (3.1)		
Academic performance				15.425	**< 0.001**				
Good	125 (52.7)	32 (36.8)	93 (62.0)			61 (55.5)	64 (50.4)	0.768	0.681
Average	57 (24.1)	25 (28.7)	32 (21.3)			26 (23.6)	31 (24.4)		
Poor	55 (23.2)	30 (34.5)	25 (16.7)			23 (20.9)	32 (25.2)		
Missing data	1	NA	NA			NA	NA		
Current smoking				2.428	0.194			0.028	0.866
Yes	6 (2.5)	4 (4.7)	2 (1.3)			3 (2.7)	3 (2.4)		
No	230 (97.5)	82 (95.3)	148 (98.7)			107 (97.3)	123 (97.6)		
Missing data	2	NA	NA			NA	NA		
Current alcohol consumption				15.765	**< 0.001**			3.828	**0.050** ^**&**^
Yes	83 (35.3)	44 (51.8)	39 (26.0)			31 (28.7)	52 (40.9)		
No	152 (64.7)	41 (48.2)	111 (74.0)			77 (71.3)	75 (59.1)		
Missing data	3	NA	NA			NA	NA		

NA, not applicable or no data available.

^#^Depressive symptoms were measured with the Center for Epidemiology Scale for Depression (CES-D), and individual with a score of 28 or higher was considered to have depressive symptoms.

${}^\psi$Chi-square tests for categorical variables and the student-*t* test for continuous variables were performed to test the differences between the two groups.

^*^P-values were derived from the Pearson’s Chi-square test, Fisher’s exact test, or *t*-test. Chi-square tests or Fisher’s exact test was used to test the difference between the depressive symptoms group and control group as well as boys and girls by categorical variables, and a *t*-test was used to test the age, BMI, and cortisol concentration difference.

^&^*P*-value of 0.05 indicated that differences showed marginal significance.

Abbreviations: *BMI* body mass index, *HSS* household socioeconomic status.

Furthermore, when stratified by sex, there were no significant differences between the groups in age, BMI, cortisol concentration, family structure, family relationship, household socioeconomic status, academic performance, or current smoking. The prevalence of having current drunk alcohol was higher among females (50.9%) than male students (40.1%), which was only borderline significant (*P* = 0.05).

### *FKBP5* methylation in cases with depressive symptoms and healthy controls

**Fig. 1:**
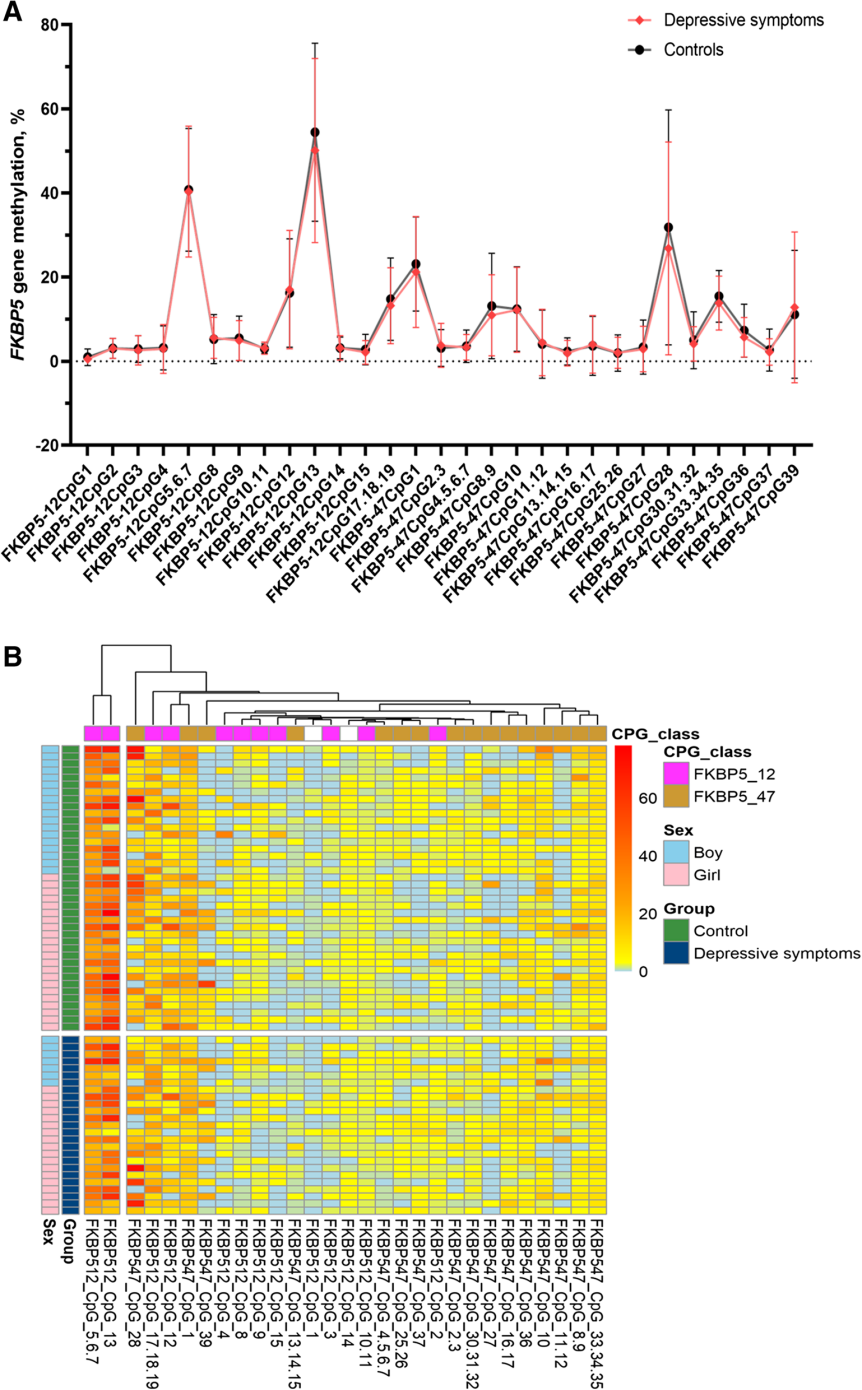
The association of *FKBP5* gene methylation, sex, and depressive symptoms. **A** Comparison of methylation degree of *FKBP5* gene between the depressive symptoms group and the healthy controls. Dots represent the mean and error bars represent the standard deviation. The red dots represent the percentage of methylation in the depressive symptoms group, whereas the black dots correspond to healthy controls. **B** The hierarchical clustering heat map of the methylation level of *FKBP5* CpG sites in adolescents with different healthy statuses (depressive symptoms and healthy controls) and sexes (boy and girl). The color key from blue to red indicates the methylation level of CpG sites from low to high, respectively. The heat map shown on the top of each dendrogram depicts the clustering of CpG sites’ grouping similarity

**Fig. 2:**
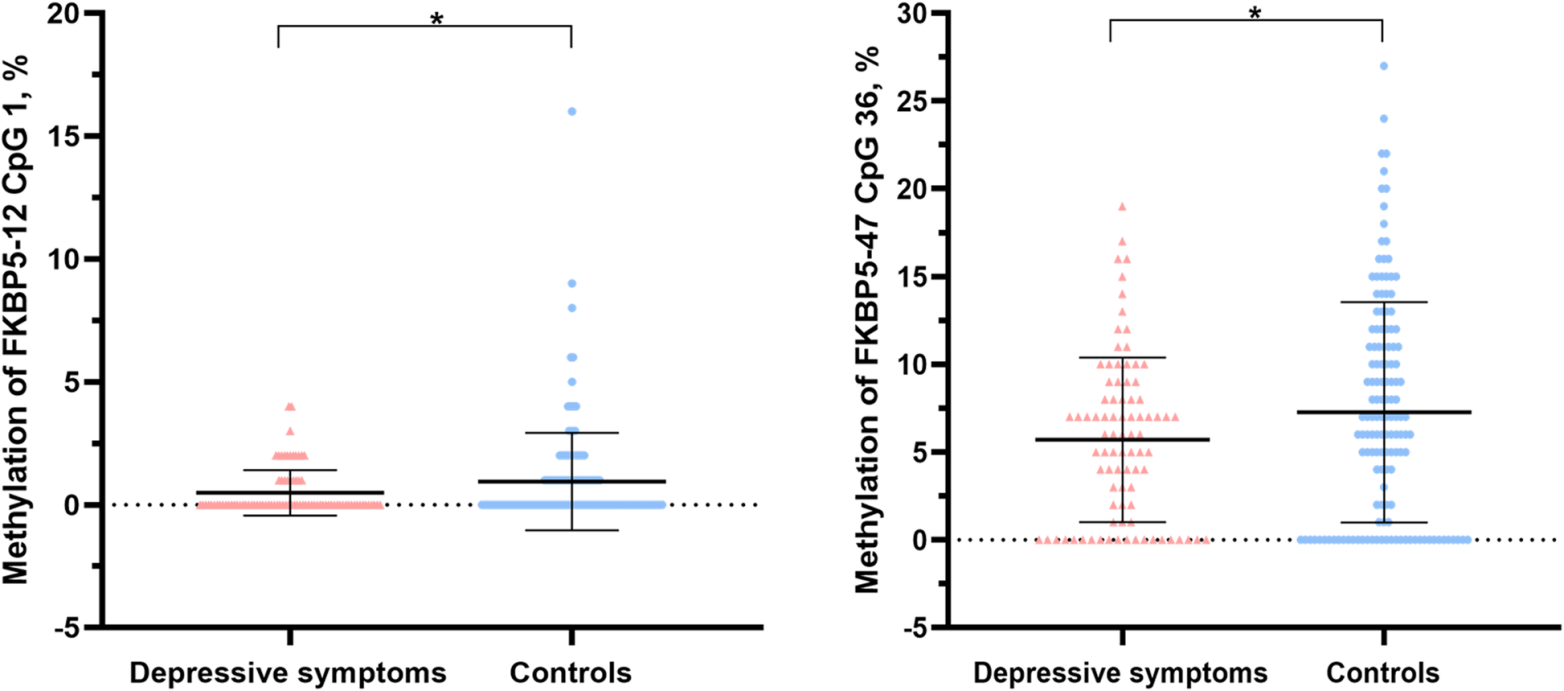
The levels of *FKBP5* methylation in adolescents with depressive symptoms and healthy controls. Student t-test was employed to compare the results between two groups. Bars represent the mean methylation level for each group, and the scatter represents the methylation level of each individual. * *P* < 0.05

The percentages of 29 CpG units encompassing 47 CpG sites in the *FKBP5* promoter region between the case group and control group were presented in a form of a line graph (Fig. 1 A). This result indicated that most of the CpG units were hypomethylation status. We also found that the overall DNA methylation trend of *FKBP5* CpG units in the persistent DS group was lower than that in the control group. Hierarchical clustering was performed using methylation percentiles per individual that showed similar methylation levels between the persistent DS group and controls (Fig. 1B). For methylation, red depicted hypermethylation and blue corresponded to hypomethylation. Of these, 6 CpG units (20.7%) presented higher methylation, and 23 CpG units (79.3%) presented lower DNA methylation levels in the *FKBP5* gene. In addition, no obvious differences in differentially methylated units between control and case groups were detected in the heatmap. Moreover, we conducted comparative methylation units between the two groups. The differences in methylation levels at two *FKBP5* CpG sites (*FKBP5*-12 CpG 1 and *FKBP5*-47 CpG 36) between the two groups were statistically significant (*P* < 0.05, Fig. 2). A further univariate analysis of covariance comparing DS group and healthy control subjects showed lower methylation levels in persistent DS group on *FKBP5*-12 CpG 1 (DS: 0.94 ± 2.00, control subjects: 0.47 ± 0.92; *F* = 5.41, *P* = 0.021). *FKBP5*-47 CpG 36 exhibited similar changes between the two groups, but the differences did not reach significance (*P* = 0.062). Correction for *FDR*, however, showed no significant differences on each CpG site for the selected markers (*q* > 0.05; detailed results were presented in Supplementary Table S1).

Subsequently, univariable logistic regression was used to screen risk factors, and then a multivariable logistic regression model was built (Table 2). The results of the univariate logistic regression analysis showed that the persistent DS were significantly related to family structure (living in a single-parent family: *OR*  =  2.96; 95% *CI*, 1.36–6.43 and living with others: *OR* = 3.05; 95% *CI*, 1.11–8.41 vs. living in a two-parent family), family relationship (average/poor: *OR* = 5.00; 95% *CI*, 2.55–9.84 vs. good), HSS (average/poor: *OR*  = 1.75; 95% *CI*, 1.03–2.99 vs. good), academic performance (average: OR = 2.20; 95% CI, 1.13–4.25 and poor: *OR* = 3.52; 95% *CI*, 1.80–6.88 vs. good) and alcohol consumption (yes: *OR* =  3.05; 95% *CI*, 1.74–5.36 vs. no). Moreover, for CpG sites, per 1% increase in methylation level at CpG sites was entered one by one in the model. The results showed that the upper limit of confidence intervals was approaching 1.00, so we cannot exclude the low methylation levels on the two CpG sites might act as risk factors in adolescents with persistent DS (*FKBP5*-12 CpG 1: *OR* = 0.79; 95% *CI*, 0.62-1.00; *P* = 0.053; *FKBP5*-47 CpG 36: *OR* = 0.95; 95% *CI*, 0.91-1.00; *P* = 0.049). Next, for multivariate logistic regression analysis, all significant factors in univariate logistic regression were included. Final multivariate model results indicated that the methylation levels of these two CpG sites did not remain statistically associated with persistent DS in the total study population (*FKBP5*-12 CpG 1: *OR* = 0.77; 95% *CI*, 0.58–1.01; *P* = 0.055; *FKBP5*-47 CpG 36: *OR* = 0.95; 95% *CI*, 0.89-1.00; *P* = 0.063). Given the above results, we were prompted to assess that a single CpG sites did not independently associate with persistent DS.

**Table 2:** Univariable and multivariable logistic regression analysis of the risk factors associated with depressive symptoms

Variables	Depressive symptoms*					
	Total		Boys		Girls	
	OR (95% CI)	*P*	OR (95% CI)	*P*	OR (95% CI)	*P*
univariable logistic regression						
Family structure						
Living in a two-parent family	1.00 (reference)		1.00 (reference)		1.00 (reference)	
Living in a single-parent family	2.96 (1.36–6.43)	0.006	2.06 (0.53–7.98)	0.295	3.14 (1.16–8.49)	0.025
Living with others	3.05 (1.11–8.41)	0.031	5.15 (1.14–23.32)	0.033	1.96 (0.49–7.77)	0.339
Family relationship						
Good	1.00 (reference)		1.00 (reference)		1.00 (reference)	
Average/Poor^&^	5.00 (2.55–9.84)	< 0.001	7.58 (2.52–22.83)	< 0.001	3.54 (1.50–8.38)	0.004
HSS						
Above average	1.00 (reference)		1.00 (reference)		1.00 (reference)	
Average/Poor^&^	1.75 (1.03–2.99)	0.040	2.85 (1.17–6.97)	0.022	1.46 (0.72–2.98)	0.293
Academic performance						
Good	1.00 (reference)		1.00 (reference)		1.00 (reference)	
Average	2.20 (1.13–4.25)	0.020	3.27 (1.18–9.03)	0.022	1.61 (0.67–3.88)	0.291
Poor	3.52 (1.80–6.88)	< 0.001	2.86 (0.99–8.29)	0.052	4.10 (1.64–10.27)	0.003
Current alcohol consumption						
No	1.00 (reference)		1.00 (reference)		1.00 (reference)	
Yes	3.05 (1.74–5.36)	< 0.001	3.09 (1.26–7.57)	0.014	2.74 (1.31–5.74)	0.007
* FKBP*5-12 CpG 1 (per 1% increase)	0.79 (0.62-1.00)	0.053	0.72 (0.47–1.11)	0.137	0.84 (0.62–1.12)	0.230
* FKBP*5-47 CpG 36 (per 1% increase)	0.95 (0.91-1.00)	0.049	0.96 (0.89–1.03)	0.284	0.96 (0.89–1.02)	0.187
multivariable logistic regression						
	aOR (95% CI)^#^	*P*	aOR (95% CI)^#^	*P*	aOR (95% CI)^#^	*P*
*FKBP*5-12 CpG 1 (per 1% increase)	0.77 (0.58–1.01)	0.055	0.64 (0.37–1.09)	0.100	0.79 (0.57–1.10)	0.169
*FKBP*5-47 CpG 36 (per 1% increase)	0.95 (0.89-1.00)	0.063	0.93 (0.84–1.02)	0.119	0.97 (0.90–1.05)	0.468

Abbreviations: *OR* odds ratio, *CI* confidence interval, *HSS* household socioeconomic status, *aOR* adjusted odds ratio.

*Depressive symptoms were measured with the Center for Epidemiological Studies Depression Scale (CES-D). A CES-D score ≥ 28 was taken as the cut-off for depressive symptoms.

^&^Because of the low number of events, poor and average family relationships or HSS were combined in the analyses.

^#^Models for depressive symptoms were adjusted for family structure, family relationship, household socioeconomic status, academic performance, and alcohol consumption, respectively.

### Associations among *FKBP5* methylation, adolescents’ sex, and depressive symptoms

To explore the sex differences in *FKBP5* methylation between cases and controls, the sex grouping was utilized in a hierarchical clustering heatmap of CpG sites methylation (Fig. 1B). No clear-cut pattern of different sexes, however, was found based on the heatmap. Results of the univariate and multivariate logistic regression after stratification by sex were presented in Table 2. The univariable logistic regression showed that boys living with others, and having average/poor family relationships, average/poor HSS, average academic performance, and alcohol consumption were more likely to suffer from persistent DS (*OR* = 2.85 ~ 7.58). Among girls, living in a single-parent family, and having average/poor family relationships, poor academic performance, and alcohol consumption were significantly related to persistent DS (*OR* = 2.74 ~ 4.10). However, regardless of sex, the association between the methylation level of two *FKBP5* CpG sites and persistent DS was not significant in univariable and multivariate logistic regression (*P* > 0.05).

## Discussion

In the present study, we evaluated the DNA methylation of the *FKBP5* gene in Chinese adolescents using a nested case-control study. We also examined whether there were sex differences in such associations. Our study indicated that there were significant differences between adolescents with persistent DS and healthy controls in family structure, family relationships, household socioeconomic status, academic performance, and alcohol consumption. The confounding effects of these variables were considered when exploring the association between DNA methylation and persistent DS. The main findings of the current research included the following. (1) Lower methylation value of *FKBP5*-12 CpG 1 in adolescents with persistent DS was observed after adjusting social-environmental covariates, although the statistical significance of the difference was lost after *FDR* correction. (2) Given the social environment influences, no independent association was detected between hypomethylation of *FKBP5* CpG sites and persistent DS. (3) No significant difference between sexes was found in these associations. Besides, these results may help us to better understand the role of *FKBP5* DNA methylation in adolescents with persistent DS.

In this study, a global reduction of *FKBP5* methylation in adolescents with persistent DS was observed, which is consistent with previous studies examining human whole-blood DNA methylation of the *FKBP5* gene in relation to depression [18, 19, 26]. A large cross-sectional study reported an inverse association between the severity of DS and methylation levels in *FKBP5*, though the relations between methylation and lifetime MDD were not found [18]. In addition, some case-control studies have investigated the correlation between epigenetic modifications in *FKBP5* and changes in structure and function in the brain, suggesting influence by childhood adversity, demethylation of *FKBP5* altered the structure of relevant brain areas and their functions predisposing MDD [19, 26].

We also included depression-related covariates in our analysis, and the results indicate that the percentage of DNA methylation at one site within *FKBP5* remained significantly lower in the persistent DS group than in the healthy control group. The univariate analyses showed that a lower methylation level of the *FKBP5* gene might be associated with persistent DS. This finding may be explained by the fact that *FKBP5* affects the GR function, thereby setting off a short negative feedback loop [41]. It has been demonstrated that the function of the GR is impaired during the development of depression due to the high expression level of *FKBP5* that leads to destabilizing the negative response and an increasing level of glucocorticoids called “glucocorticoid resistance” [42]. Glucocorticoids overexpression reduces neurogenesis and synaptogenesis, and causes higher emotional lability directly, which are sensitive to the onset of mental disorders [43]. However, the differences in methylation levels at *FKBP5* CpG site between the two groups were no longer statistically significant after *FDR* correction, which could be due to the following reasons. A possible reason is that our sample size may not be large enough to detect minimal associations and further research on a larger sample size is needed to confirm these results. Another possible explanation for this might pertain to the characteristics of the study population. Depressive symptom severity was relatively mild in our school-based sample compared to clinical samples. Hence, we might have missed the effects of severe depression.

The univariable logistic regression analyses also found that adolescents living with a single parent or other relatives, coming from poor relationships and economic status families, were more likely to have persistent DS. In addition, those who had poor academic performance or drinking were also at higher risk of developing more DS. These also accorded with our earlier observations [29]. Further multivariate logistic regression analysis, after adjustment for the above risk factors, showed that there was no independent association between hypomethylation of *FKBP5* CpG sites and persistent DS.

Depression is a complex disease involving multiple genetic, epigenetic, and environmental alterations. Our results were consistent with previous reports [23–25]. Weder et al. [23] in a case-control study, conducted genome-wide methylation research among 190 children and also discovered that children’s depression scores were significantly predicted by methylation in CpG sites of *FKBP5*, but did not reach statistical significance after correcting for whole genome testing. A separate longitudinal study on this issue by Humphreys et al. [25] also suggested that DNA methylation levels of CpG sites within *FKBP5* did not predict the onset of MDD in 77 adolescent girls. Similar to these studies reporting no associations, our participants were adolescents ages 12 to 17, drawing from a school-based, population-representative cohort, while other studies were mostly adult participants and MDD patients that suggested more severe DS compared to school samples.

In contrast to our findings, methylation levels have been reported to be elevated in MDD in other studies [20, 21]. Nevertheless, the research subjects in these studies were MDD patients with serious suicidal ideation or remitted MDD patients, which could lead to substantially different results, and the potential impact of social environment covariates was not taken into account. Although Roy et al. [21] reported a significant increase in DNA methylation of the *FKBP5* promoter region in MDD patients, effects might be only carried by the patients accompanied by serious suicidal ideation. In another study, Höhne et al. [20] showed a non-significant trend of increased methylation in remitted patients with MDD compared to healthy controls. Knowledge about epigenetic modifications of the gene is constantly updated, however, the clinical significance of hypermethylation remains unknown.

As part of an exploratory analysis, we analyzed the sex differences in the association of methylation status with persistent DS. The results of the present study showed that girls had more depressive tendencies than boys, which is in line with the findings of many previous studies [3, 4]. The ABC (affective, biological, cognitive) model suggests that multiple factors contribute to the sex differences in depression, and any single pathway can only partly explain the variation in depression [44]. The univariate analyses in our study suggested that significant sex differences were detected in the association between environmental risk factors and persistent DS, but not in the association between hypomethylation of *FKBP5* and persistent DS in the currently studied sample size. The influence of sex on methylation in depression was also reported by Xia et al. [27]. They using data of 1408 human postmortem brain samples, demonstrated that sex-differential DNA methylation with its regulatory networks had a contribution to the risks of psychiatric disorders, such as autism spectrum disorder, schizophrenia, and MDD. They found hypomethylation in the female with MDD at four CpG sites on *DUSP6* genes, however, similar results were not observed in the *FKBP5* gene. Nevertheless, the sex differences are intriguing, and more research is still needed in the future to explore the specific mechanisms at play.

To the best of our knowledge, relatively few studies have explored the association between *FKBP5* methylation levels and persistent DS in Chinese adolescents. A strength of our study is the epidemiological perspective that highlights these associations observed herein. To better identify the role of DNA methylation, our study considered not only sociodemographic variables but also health-related behaviors at baseline as covariates. Meanwhile, we conducted a preliminary exploration of sex differences in epigenetic alterations, while most studies did not consider such differences.

The current study also had several limitations. First, the study was limited by the modest sample size, which might not be large enough to detect small differences. Second, we only explored the methylation level of *the FKBP5* promoter region and did not contain other regions of the gene, such as introns 2 and 7, though the function of the promoter region is also closely related to depression [22]. Third, the relationships between gene expression levels and polymorphisms with DS has historically been lacking with respect to available evidence. Finally, the current investigation reports the DNA methylation levels in the blood, which may differ from methylation patterns in other tissues like the brain. There is emerging research, however, suggesting that DNA methylation in the blood biospecimens may serve as a systemic marker and substitute for methylation in brain tissue [45, 46]. In future research, multi-gene effects on the HPA axis signal pathway and environmental stressors, especially adverse early experiences, should be considered towards a better understanding of gene-environment interactions.

## Conclusion

In conclusion, we found a lower methylation value of *FKBP5*-12 CpG 1 in adolescents with persistent DS, although the difference was not significant after correction for multiple testing. In addition, the hypomethylation of *FKBP5* CpG sites did not independently associate with persistent DS after adjusting for social-environmental factors. Moreover, we did not observe significant sex differences in the examined analysis herein. Taken together, our findings contribute to a better understanding of complex mechanisms involving interactions of both gene and environmental factors involved in depression. Future studies are encouraged to investigate environmental, genetic, epigenetic factors, and their interaction in depression through prospective studies.

## Supplementary information

Additional file 1. Supplement

## Data Availability

The datasets used and/or analyzed during the current study are available from the corresponding author on reasonable request.

## Abbreviations

FKBP5: FK506 binding protein 5
DS: Depressive symptoms
MDD: Major depressive disorder
CpGs: CpG islands
GR: Glucocorticoid receptor
HPA: Hypothalamus-pituitary-adrenocortical
LSAMBR: Longitudinal study of adolescents’ mental and behavioral well-being research
CES-D: Center for epidemiological studies depression scale
SAP: Shrimp alkaline phosphatase
OR: Odds ratio
CI: Confidence interval
HSS: Household socioeconomic status
aOR: Adjusted odds ratio
