# Supplementary material for: The association of FKBP5 gene methylation, adolescents’ sex, and depressive symptoms among Chinese adolescents: a nested case-control study
Source: BMC Psychiatry. 2022 Nov 30;22:749. doi: 10.1186/s12888-022-04392-2 (PMC9710023; doi:10.1186/s12888-022-04392-2)
Supplement: Supplementary file 1 — Supplement [file 12888_2022_4392_MOESM1_ESM.docx]

**Supplement**

**Primer information:**

The putative CpG islands of the *FKBP5* gene were analyzed in an upstream 5000 bp to the downstream 1000 bp sequence of the TSS using the CpG islands searcher (http://www.ebi.ac.uk/Tools/seqstats/emboss_cpgplot/). The primer was designed by Agena EpiDesigner, and the primers of CpG islands in the promoter of the *FKBP5* gene were presented in **Figure S1-S2**.


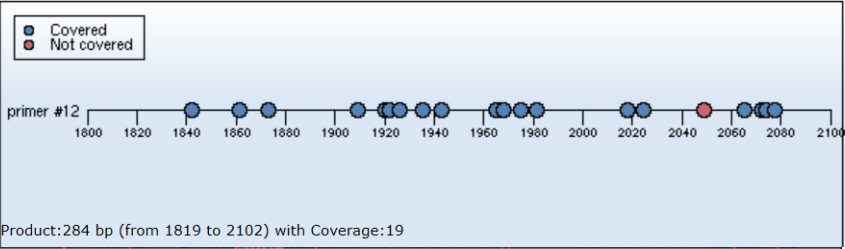


**Supplementary Figure 1.** The position of the DNA fragment corresponding to primer #12

Note: Each dot in the figure indicated one CpG site: the blue dots indicated covered CpG sites, and the red dot indicated uncovered CpG sites. 19 out of 20 CpG sites in this sequence are detected in our study.

Forward PCR primer:

5’ - aggaagagagGTTGGAGAGGGTTTATTGGGAGT - 3’ (lower case letters indicate T7 primers)

Reverse PCR primer:

5’- cagtaatacgactcactatagggagaaggctCCCCAATTACCTTAACATCAATAAC - 3’ (lower case letters indicate T7 primers)


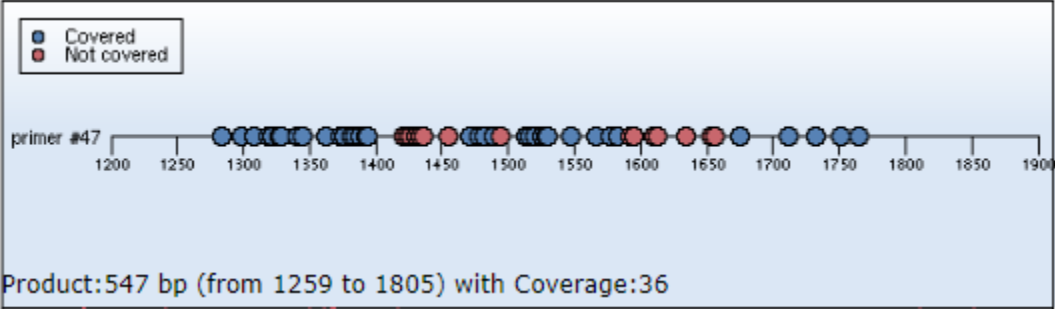


**Supplementary Figure 2.** The position of the DNA fragment corresponding to primer #47

Note: Each dot in the figure indicated one CpG site: the blue dots indicated covered CpG sites, and the red dot indicated uncovered CpG sites. 36 out of 51 CpG sites in this sequence are detected in our study.

Forward PCR primer:

5’ - aggaagagagGAGATTTTGAGAATTTGGGTTTAG - 3’ (lower case letters indicate T7 primers)

Reverse PCR primer:

5’ - cagtaatacgactcactatagggagaaggctACTTCCCTTACAAAAAACCAAAAAA - 3’

(lower case letters indicate T7 primers)

| **Table S1.** Association of *FKBP5* gene CpG sites methylation with depressive symptoms at follow-up. | | | | | |
| --- | --- | --- | --- | --- | --- |
| CpG sites | Total methylation level | Depressive symptoms | Controls | *P* ^*^ | *q* ^#^ |
|  | (mean ± SD, %) | (mean ± SD, %) | (mean ± SD, %) |  |  |
| ***FKBP5*-12 CpG 1** | **0.77 ± 1.68** | **0.48 ± 0.93** | **0.94 ± 1.98** | **0.019** | 0.493 |
| *FKBP5*-12 CpG 2 | 3.04 ± 2.37 | 3.05 ± 2.39 | 3.04 ± 2.37 | 0.989 | 0.990 |
| *FKBP5*-12 CpG 3 | 2.78 ± 3.29 | 2.59 ± 3.47 | 2.89 ± 3.19 | 0.498 | 0.903 |
| *FKBP5*-12 CpG 4 | 3.08 ± 5.43 | 2.91 ± 5.78 | 3.18 ± 5.22 | 0.725 | 0.914 |
| *FKBP5*-12 CpG 5.6.7 | 40.58 ± 14.92 | 40.32 ± 15.56 | 40.74 ± 14.57 | 0.838 | 0.972 |
| *FKBP5*-12 CpG 8 | 5.38 ± 5.49 | 5.57 ± 4.88 | 5.26 ± 5.84 | 0.669 | 0.914 |
| *FKBP5*-12 CpG 9 | 5.25 ± 5.08 | 4.91 ± 4.75 | 5.45 ± 5.28 | 0.435 | 0.901 |
| *FKBP5*-12 CpG 10.11 | 3.02 ± 1.26 | 3.09 ± 1.44 | 2.97 ± 1.14 | 0.486 | 0.903 |
| *FKBP5*-12 CpG 12 | 16.53 ± 13.32 | 17.03 ± 14.07 | 16.22 ± 12.89 | 0.681 | 0.914 |
| *FKBP5*-12 CpG 13 | 52.83 ± 21.48 | 50.10 ± 21.88 | 54.45 ± 21.16 | 0.138 | 0.800 |
| *FKBP5*-12 CpG 14 | 3.16 ± 2.69 | 3.16 ± 2.90 | 3.17 ± 2.57 | 0.990 | 0.990 |
| *FKBP5*-12 CpG 15 | 2.53 ± 3.35 | 2.10 ± 2.80 | 2.78 ± 3.63 | 0.115 | 0.800 |
| *FKBP5*-12 CpG 17.18.19 | 14.17 ± 9.51 | 13.20 ± 9.02 | 14.75 ± 9.78 | 0.228 | 0.857 |
| *FKBP5*-47 CpG 1 | 22.40 ± 11.99 | 21.21 ± 13.18 | 23.12 ± 11.19 | 0.245 | 0.857 |
| *FKBP5*-47 CpG 2.3 | 3.39 ± 4.70 | 3.76 ± 5.21 | 3.16 ± 4.36 | 0.356 | 0.857 |
| *FKBP5*-47 CpG 4.5.6.7 | 3.46 ± 3.58 | 3.30 ± 3.06 | 3.55 ± 3.86 | 0.616 | 0.914 |
| *FKBP5*-47 CpG 8.9 | 12.27 ± 11.53 | 10.93 ± 9.64 | 13.12 ± 12.55 | 0.173 | 0.836 |
| *FKBP5*-47 CpG 10 | 12.32 ± 10.05 | 12.22 ± 10.12 | 12.39 ± 10.05 | 0.911 | 0.990 |
| *FKBP5*-47 CpG 11.12 | 4.19 ± 8.01 | 4.45 ± 7.91 | 4.06 ± 8.10 | 0.765 | 0.924 |
| *FKBP5*-47 CpG 13.14.15 | 2.16 ± 3.16 | 1.90 ± 3.05 | 2.31 ± 3.23 | 0.346 | 0.857 |
| *FKBP5*-47 CpG 16.17 | 3.78 ± 6.93 | 4.00 ± 6.87 | 3.65 ± 6.99 | 0.715 | 0.914 |
| *FKBP5*-47 CpG 25.26 | 1.95 ± 4.07 | 1.99 ± 3.69 | 1.93 ± 4.31 | 0.928 | 0.990 |
| *FKBP5*-47 CpG 27 | 3.15 ± 6.02 | 2.88 ± 5.40 | 3.34 ± 6.43 | 0.631 | 0.914 |
| *FKBP5*-47 CpG 28 | 29.76 ± 26.87 | 26.83 ± 25.29 | 31.82 ± 27.92 | 0.331 | 0.857 |
| *FKBP5*-47 CpG 30.31.32 | 4.66 ± 5.88 | 4.12 ± 4.08 | 4.99 ± 6.75 | 0.289 | 0.857 |
| *FKBP5*-47 CpG 33.34.35 | 14.82 ± 6.28 | 13.81 ± 6.41 | 15.43 ± 6.15 | 0.061 | 0.590 |
| ***FKBP5*-47 CpG 36** | **6.67 ± 5.77** | **5.70 ± 4.68** | **7.26 ± 6.28** | **0.034** | 0.493 |
| *FKBP5*-47 CpG 37 | 2.48 ± 4.38 | 2.19 ± 3.12 | 2.66 ± 5.00 | 0.384 | 0.857 |
| *FKBP5*-47 CpG 39 | 11.81 ± 16.30 | 12.80 ± 17.90 | 11.14 ± 15.19 | 0.541 | 0.914 |
| ^*^ *P*-values were derived from *t*-test for depressive symptoms.  ^#^ Adjusted *P*-values after *t*-tests with *FDR* correction. | | | | | |
